# Disruption of mesoderm formation during cardiac differentiation due to developmental exposure to 13-*cis*-retinoic acid

**DOI:** 10.1038/s41598-018-31192-0

**Published:** 2018-08-28

**Authors:** Qing Liu, Kevin Van Bortle, Yue Zhang, Ming-Tao Zhao, Joe Z. Zhang, Benjamin S. Geller, Joshua J. Gruber, Chao Jiang, Joseph C. Wu, Michael P. Snyder

**Affiliations:** 1 Department of Genetics, Stanford University School of Medicine, Stanford, California, 94305 USA; 2 Genetics Bioinformatics Service Center, Stanford University School of Medicine, Stanford, California, 94304 USA; 3 Stanford Cardiovascular Institute, Stanford University School of Medicine, Stanford, California, 94305 USA; 4 Department of Medicine, Oncology Division, Stanford University School of Medicine, Stanford, California, 94305 USA

## Abstract

13-*cis*-retinoic acid (isotretinoin, INN) is an oral pharmaceutical drug used for the treatment of skin acne, and is also a known teratogen. In this study, the molecular mechanisms underlying INN-induced developmental toxicity during early cardiac differentiation were investigated using both human induced pluripotent stem cells (hiPSCs) and human embryonic stem cells (hESCs). Pre-exposure of hiPSCs and hESCs to a sublethal concentration of INN did not influence cell proliferation and pluripotency. However, mesodermal differentiation was disrupted when INN was included in the medium during differentiation. Transcriptomic profiling by RNA-seq revealed that INN exposure leads to aberrant expression of genes involved in several signaling pathways that control early mesoderm differentiation, such as TGF-beta signaling. In addition, genome-wide chromatin accessibility profiling by ATAC-seq suggested that INN-exposure leads to enhanced DNA-binding of specific transcription factors (TFs), including HNF1B, SOX10 and NFIC, often in close spatial proximity to genes that are dysregulated in response to INN treatment. Altogether, these results identify potential molecular mechanisms underlying INN-induced perturbation during mesodermal differentiation in the context of cardiac development. This study further highlights the utility of human stem cells as an alternative system for investigating congenital diseases of newborns that arise as a result of maternal drug exposure during pregnancy.

## Introduction

Congenital heart defects (CHDs) are the most common form of birth defects, with nearly 1% of newborns (about 35,000–40,000) affected with CHDs every year^1,2^. All-*trans* retinoic acids (RAs) have been shown to play important roles in organizing embryonic development through multiple signaling pathways^3^. However, 13-*cis*-RA, known as isotretinoin (INN) and used for the treatment of acne, is classified as a pregnancy category X drug by the FDA and is correlated with a high risk of birth defects. INN is therefore considered to be a teratogen and is prohibited for use by pregnant women^4,5^. It has been reported that administration of INN during pregnancy causes multiple types of malformations in infants, including cardiovascular, craniofacial, thymic, and neural developmental defects^6–10^. Unlike other RAs, INN exhibits low affinity for both RA receptors (RARs, for all-trans RA) and retinoid X receptors (RXRs, for 9-cis retinoic acid)^4^. Although INN has been widely used for dermatological therapy, its cellular and molecular mechanisms underlying developmental toxicity still remain poorly understood.

Human induced pluripotent stem cells (hiPSCs) and human embryonic stem cells (hESCs) provide a valuable opportunity for the investigation of human diseases and drug screening. Compared to other animal models, human stem cells avoid species-specific effects that may arise during the use of non-human model organisms. Human stem cells can be differentiated into multiple types of cells, serving as organoids for specific disease models; therefore, this system obviates the limitations of using immortalized transformed cell lines (e.g., HepG2 and HEK293)^11–13^. For example, differentiation of cardiomyocytes derived from hiPSCs from clinical patients has been used to study cardiac diseases and to evaluate drug-induced cardiotoxicity in hiPSC-derived cardiomyocytes^14–17^. However, application of human stem cells to research in developmental toxicology (i.e., cells exposed to drugs during differentiation) has been rarely documented.

The objective of the present study was to elucidate the molecular mechanism(s) underlying INN-induced perturbation during early differentiation stages using human stem cells as an *in-vitro* model system. To accomplish this, we exposed both hESCs and hiPSCs to a sublethal concentration of INN during cardiomyocyte differentiation. RNA sequencing (RNA-seq) and assay of transposase-accessible chromatin with high-throughput sequencing (ATAC-seq) were performed to determine the transcription factors (TFs) and gene-expression regulatory mechanism(s) linked to INN-induced perturbations at early stages of differentiation. We observed that INN specifically inhibited mesodermal differentiation. Integrative analyses of RNA-seq and ATAC-seq data revealed that INN-exposure caused dysregulation of genes involved in multiple signaling pathways important for mesodermal development.

## Results

### Developmental INN exposure inhibits mesoderm formation during cardiac differentiation

**Figure 1:**
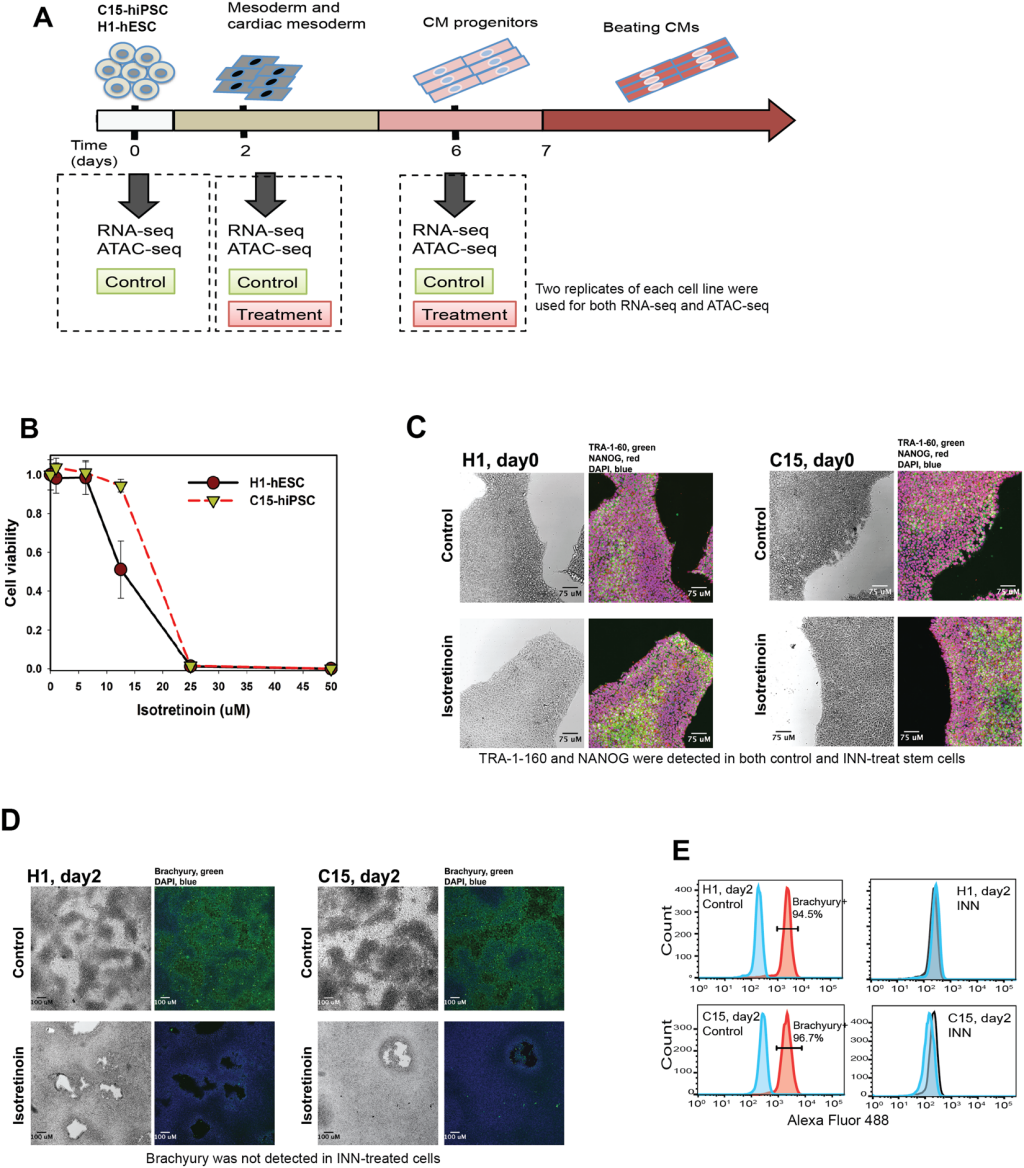
INN-induced disruption of mesoderm formation during early cardiac differentiation. (**A**) Experimental design. Cardiomyocyte differentiation from both C15-hiPSCs and H1-hESC. Isotretinoin at a concentration of 25 nM was added to the medium during the differentiation process. Cells from two replicates of each line were collected on day 2 (mesoderm) and day 6 (cardiomyocyte progenitor) for RNA-seq and ATAC-seq. Undifferentiated stem cells on day 0 were collected and used as a reference. Two replicates of each cell line were used for RNA-seq and ATAC-seq. (**B**) Cell viability upon exposure to Isotretinoin for 48 h. (**C**) INN-exposure at 25 nM does not influence the pluripotency of the stem cells Immunostaining of stem cells (day 0) with TRA-1-60 (green) and NANOG (red), the nuclei were stained with DAPI (blue). No difference was observed between control and the INN-treated group. Both TRA-1-60 and NANOG were detected. (**D**–**E**) INN inhibits mesoderm formation during cardiac differentiation. Immunostaining of mesoderm cells with Brachyury (green) on day 2. No obvious Brachyury-expressed cells were found in the INN-treated cells by fluorescent microscopy (**D**) and flow cytometry analysis (**E**).

In the present study, we performed a monolayer-differentiation method to differentiate both hiPSCs and hESCs into cardiomyocytes as we have previously described^18^. Intermediate stages of cardiomyocyte differentiation include mesoderm formation (day2), cardiac mesoderm (days 4–5), and cardiomyocyte progenitors (day 6)^18^; finally, beating cardiomyocytes are observed after 7–10 days (Fig. 1A, and Supplementary videos 1 and 2).

Two steps of experiments were performed to determine the lowest-observed-adverse-effect level (LOAEL) of INN for this study. At the first step, dose-response experiments were performed to determine a sublethal concentration of INN for undifferentiated stem cells; at the second step, stem cells were exposed to a wide range of concentrations of INN identified from the first step during cardiomyocyte differentiation, so as to determine a LOAEL of INN, which does not impair the process of cardiac differentiation. Specifically, dose-response experiments (0–50 μM) were performed to determine a sublethal concentration of INN for undifferentiated stem cells for 48 h. We chose 48 h as the differentiation medium was replaced every 48 h in the subsequent differentiation experiments. The doses-response curves of stem cells exposed to INN for 48 h are shown in Fig. 1B; both cell lines did not show mortality at concentrations below 5 μM. Moreover, pre-exposure to INN did not influence the self-renewal and proliferation of hESCs and hiPSCs, and the expression of pluripotency marker NANOG and surface marker TRA-1-60 were appropriately observed in the pre-exposed stem cells (Fig. 1C). The INN-exposed stem cells can still be differentiated to beating cardiomyocytes after removal of INN at the initiation of the differentiation (Supplementary videos 3 and 4).

Subsequently, we exposed both C15-hiPSCs and H1-hESCs to a wide range of sublethal concentrations of INN (from 1 nM to 5 μM) during cardiomyocyte differentiation, all of which appeared to be non-toxic in these stem cells. We further observed that cellular morphology remained unchanged on day 2 after initiation of differentiation (mesoderm stage) when the concentration exceeded 25 nM. On day 2, the control cells entered the mesoderm stage and highly expressed the Brachyury protein (Fig. 1D), a mesodermal marker encoded by gene *T*. In contrast, Brachyury was not observed in the INN-treated group, which exhibited a flatter colony surface. The INN-treated cells also exhibited a much smaller and denser morphology in the plates compared to the mesoderm cells in the control group (Fig. 1D,E). On day 6, the cells were differentiated into cardiomyocyte progenitors, and the cardiac markers NKX2-5 and TNNT2 were detected in the control group (Supplementary Figure 1). After day 7, early beating cardiomyocytes were observed in the control group (Supplementary videos 1 and 2). However, cellular morphologic development remained nearly static in the INN-treated group during the differentiation after day 2, with only minimal detection of TNNT2 (Supplementary Figure 1).

In addition, we did not observe perturbation in cardiac differentiation if INN was added to the medium after mesoderm formation (data not shown), suggesting the detrimental effects of INN exposure are most pronounced during mesodermal differentiation. We also tested the effects of INN exposure on endoderm and ectoderm formation, and observed that 25 nM INN had minor effects on ectoderm formation and moderate effects on endoderm formation, respectively (Supplementary Figures 2 and 3). Based on the flow-cytometry analysis, ectodermal cells were decreased 3% in C15-iPSCs and endodermal cells were decreased to about 27.5% in both C15-iPSCs and H1-hESCs compared to those in control (Supplementary Figure 3). Collectively, these results suggested that INN exposure exerted much more deleterious effects on mesodermal formation-especially during cardiac lineage differentiation.

Based on these results, we established 25 nM as the LOAEL of INN for disruption of mesoderm formation in this study. It is worth noting that this concentration is substantially lower than the average steady-state plasma concentration observed in patients (~670 nM)^19^. We next explored the transcription and chromatin accessibility patterns in stem cells upon exposure to 25 nM INN to gain new insight into the potential mechanisms driving INN-induced disruption during mesoderm formation derived from C15-iPSCs and H1-hESCs.

### INN-exposure induces dysregulation of genes related to mesoderm differentiation during cardiomyocyte differentiation

**Figure 2:**
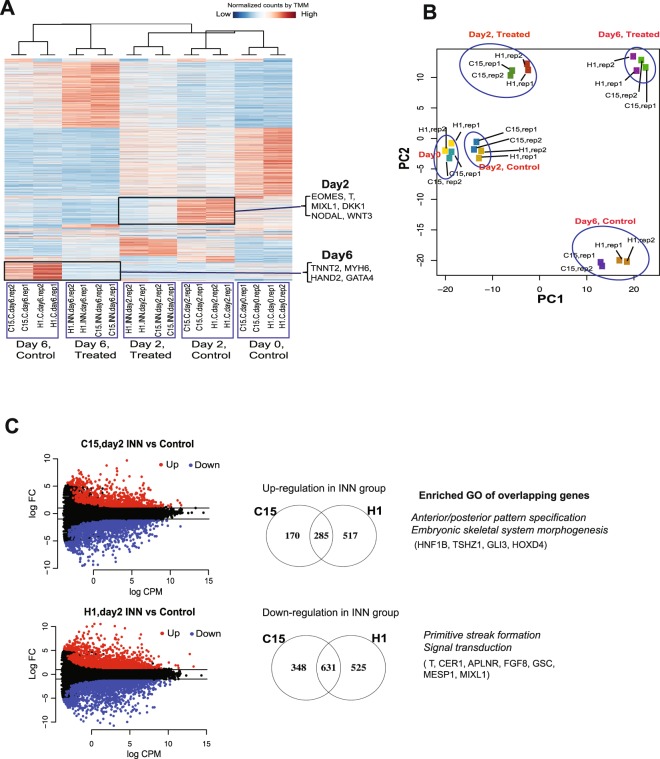
Analysis of genome-wide transcriptome of control and INN-treated groups during cardiac differentiation from day 0 to day 6. (**A**) Clustering analysis of transcriptomic profiles clustered by weighted gene co-expression network analysis (WGCNA). The heatmap represents the normalized counts by trimmed mean of M-values (TMM) normalization method. Representative dysregulated genes are shown, such as down-regulation of *EOMES*, *T* and *NODAL* in INN-treated cells. **(B)** PCA analysis of samples based upon their genome-wide transcriptomic profiles. **(C)** Left, MA-plot of the log-fold change against log-counts per million (log-CPM) of all genes. The differentially up- and down-expressed genes (FDR < 0.05) in INN-treated cells were labeled with red and blue, respectively. Right, Correlation plot of gene expression in INN-treated cells at day 2 between C15 and H1. The representatively top up- and down-regulated genes are listed in the figure. Statistically enriched gene ontology (GO) (*p* < 0.05) of up-regulated genes between C15 and H1 are related to anterior/posterior pattern specification and embryonic skeletal system morphogenesis, and down-regulated genes are related to primitive streak formation and signal transduction.

Identifying changes in transcription between control and drug-treatment may help provide insight into how INN-exposure disrupts mesoderm formation. Global gene expression patterns were therefore profiled longitudinally at days 0, 2, and 6 by RNA-seq experiments in both control and INN-treated stem cells (Fig. 1A). Weighted gene co-expression network analysis (WGCNA), which calculates correlation values between individual gene expression profiles, was performed across these samples^18,20^. Overall, transcription patterns clearly cluster by time point and treatment condition. Furthermore, both C15 and H1 cells exhibit similar expression patterns in control and treatment groups (Fig. 2A), indicating that changes in transcription induced by INN exposure are largely concordant between H1 and C15 cell lines.

Further analysis reveals specific clusters of genes that respond differently between control and INN-treatment groups during differentiation. For example, several genes known to regulate mesoderm formation and cardiac differentiation were highly expressed in control groups compared to the INN-treated groups on days 2 and 6, respectively (Fig. 2A). These genes included *EOMES*, *T*, *MIXL1*, *DKK1*, *NODAL* and *WNT3* on day 2; and *TNNT2*, *MYH6*, *HAND2* and *GATA4* on day 6 (Fig. 2A and Supplementary Table 1). The same pattern of sample segregation was also observed by principal component analysis (PCA) (Fig. 2B). As one might expect, the enriched GO terms (biological process, BP) on day 2 included early mesoderm differentiation features, such as “pattern specification process”, “anterior/posterior pattern formation” and “embryonic morphogenesis” for both untreated H1 and C15 cell lines (Supplementary Table 2). On day 6, the GO term of “heart development” was appropriately enriched in control cells (*i*.*e*., cardiomyocyte progenitors) (Supplementary Table 2). However, these developmental stage-specific GO terms (*e*.*g*., embryonic morphogenesis) were not associated with the up-regulated genes in the treatment groups (Supplementary Table 3).

The differential genes (FDR <0.05) on day 2 exhibited similar changes in both H1 and C15 lines (Pearson correlation coefficient 0.92). Genes commonly upregulated in H1 and C15 lines are related to “anterior/posterior pattern specification” and “embryonic skeletal system morphogenesis”, whereas genes commonly downregulated are related to “primitive streak formation” and “signal transduction” (Fig. 2C and Supplementary Table 4), suggesting genes involved in early developmental processes and signal transduction are aberrantly expressed in response to INN treatment at this early time point.

### Transcription factor footprinting analysis reveals dynamic occupancy at specific regulatory elements after INN-exposure

**Figure 3:**
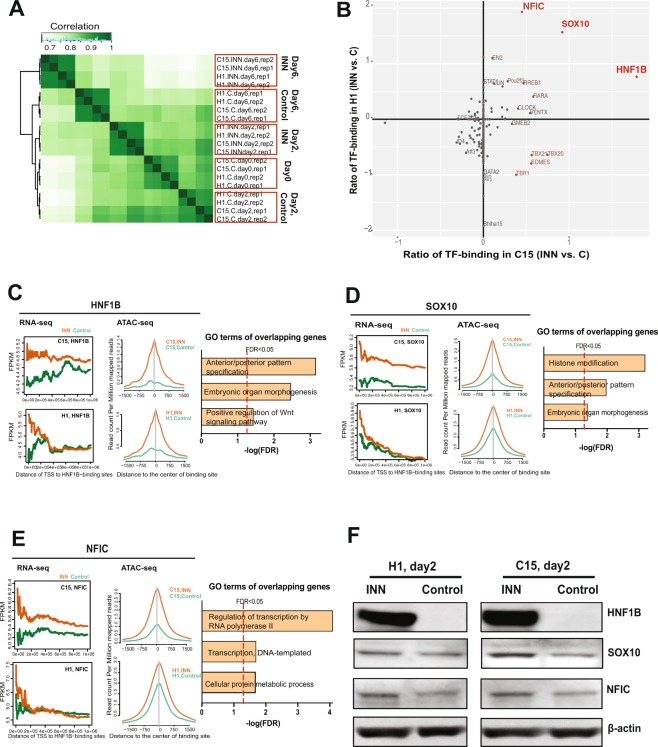
Analyses of genome-wide chromatin landscape and enhanced TF-binding events in INN-treated groups during mesoderm differentiation. (**A**) Correlation of differential peaks in control and INN-treated groups of two cell lines during cardiomyocyte differentiation. (**B**) Median differential chromatin accessibility at TF motifs independently identified across H1 and C15 cell lines. HNF1B, SOX10, and NFIC show enhanced footprint accessibility in INN-treated cells on day2. (**C**–**E**) Left, Plotting of expression values (i.e., fragments per kilobase of transcript per million mapped reads [FPKMs]) of differential genes in close spatial proximity to the genomic binding sites of these TFs (specifically, within a moving window of 1500-bp distance to the transcription start site [TSS]). Middle, ATAC-seq signals (i.e. open chromatin peaks) around the center of TF-binding sites across the whole genome in the treatment group is higher than that in control. Right, Top enriched gene ontology (GO) terms of the overlapping genes that were likely regulated by the TFs in both H1 and C15. The red dotted line denotes statistical significance (FDR <0.05) (F) Protein expression of HNF1B, SOX10, and NFIC in INN-treated and control cells on day2 using Western blot analysis. These TFs showed an up-regulation in INN-treated cells compared to that in control. Full-length blots of these cropped images are presented in the Supplementary Figure 7.

To better understand the molecular determinants driving aberrant expression patterns upon INN treatment, we next profiled the regulatory chromatin landscape using ATAC-seq across each time-point and condition. Hierarchical clustering of genome-wide chromatin accessibility profiles revealed a strong correlation pattern in the global chromatin landscape between cell lines at each time-point (Fig. 3A), as similarly observed for transcription patterns (Fig. 2A). These results suggest that hESC and hiPSC cell lines show similar patterns of chromatin accessibility specific to INN exposure.

Beyond the general identification of open chromatin sites, ATAC-seq read ends can be exploited to identify TF motifs that are protected by protein-DNA interaction. This information can then be used to determine the likelihood of TF binding at a given regulatory element, which may provide insight into which factors are driving dynamic transcription patterns. We therefore identified transcription factor genome-wide footprints using the Protein Interaction Quantification (PIQ) footprinting algorithm^21^ against a curated motif database (http://jaspar.genereg.net)^22^, filtering with a positive predictive value (PPV) cutoff of 0.7. Changes in chromatin accessibility were queried across putative TF binding sites (specifically their distance to the transcription start sites [TSS]) as a proxy for changes in regulatory element occupancy, and they were then compared with dynamic transcription patterns observed between control and INN-exposure conditions. We leveraged results from this combinatorial approach to identify potential regulatory mechanisms underlying INN-induced transcriptional disruption during mesoderm formation.

Comparison of TF footprint accessibility between treatment and control conditions (*i*.*e*. treatment *vs*. control) revealed that specific TFs showed enhanced overall chromatin accessibility at binding events after exposure to INN in both H1 and C15 lines, including HNF1 Homeobox B (HNF1B), SRY-Box 10 (SOX10) and Nuclear Factor I C (NFIC) (Fig. 3B). The differential accessibility scores of each TF footprint are shown in Supplementary Table 5. Further examination of genes that are proximal to HNF1B, SOX10, and NFIC binding sites revealed strong higher average FPKM values in the INN-exposure group, particularly for genes in which the TSS is immediately adjacent to the bound TF motif (Fig. 3C–E, further details provided in Supplementary Table 6). GO enrichment analysis revealed these proximal genes to be related to “anterior/posterior pattern specification”, “embryo organ morphogenesis”, and “regulation of by RNA polymerase II” in both H1 and C15 cell lines (Fig. 3C–E). Furthermore, inspection of aggregate chromatin accessibility in control and INN-treated cells further illustrates the higher signal intensity surrounding the footprints identified for these TFs (Fig. 3C–E), demonstrating the strong correlation between dynamic gene expression and occupancy by these TFs. Importantly, analysis of protein expression levels also revealed a significant spike in HNF1B protein specifically upon INN exposure. We also observed modest increases in protein levels of both SOX10 and NFIC (Fig. 3F), and changes in protein levels for these transcription factors are concordant with up-regulated mRNA expression levels upon INN exposure (Supplementary Figure 4). In combination with transcription and chromatin accessibility results, these results support a potential role for these factors in underlying aberrant expression patterns specific to INN exposure. Altogether, these results implicate HNF1B, SOX10, and NFIC as important transcriptional regulatory factors involved in the disruption of mesoderm formation.

### Disruptions in signaling pathways due to INN exposure during mesoderm formation

**Figure 4:**
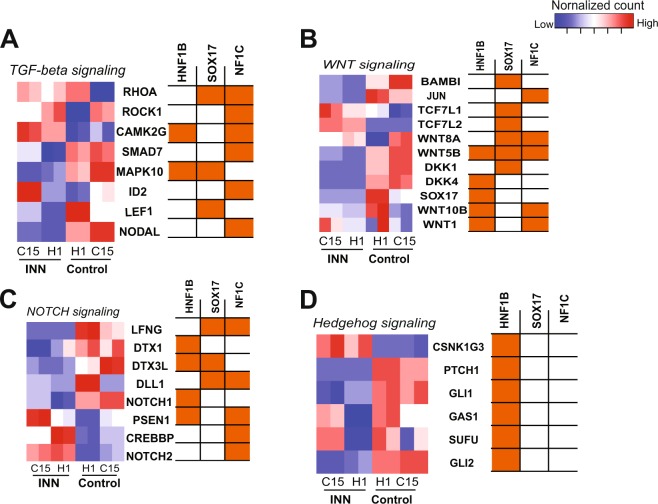
Signaling pathway analysis. (**A**–**D**) Heatmap represents expression of overlapping dysregulated of genes involved in multiple signaling pathways upon exposure to INN in both H1 and C15 cells on day2, including **(A)** TGF-beta signaling, **(B)** Wnt signaling, **(C)** Notch signaling, and **(D)** Hedgehog signaling. Right, the correlation between predicted target genes and the TFs based on footprinting analysis, *i*.*e*., correlation between transcription start site (TSS) and the bound TF motif. The cells labeled with orange means the genes were predicted to be likely regulated by the TFs.

The enrichment of GO terms related to early embryonic development and signaling pathways among INN-disrupted genes (Supplementary Tables 5 and 7) suggested to us that INN might primarily inhibit mesodermal development by altering the expression of genes involved in signaling pathways that drive normal mesoderm formation. We therefore explored the expression of several sets of genes involved in multiple pathways to gain additional insights into the INN-induced disruption of signaling pathways during mesodermal differentiation from both cell lines. Relevant signaling pathways included Wnt/β-catenin, TGF-beta, NOTCH, and Hedgehog signaling, which are all known to regulate mesodermal differentiation^23–29^. Indeed, aberrant expression of genes related to these pathways, such as *NOTCH1*, *NODAL and WNT5B*, was observed in INN-treated samples compared to that in control (Fig. 4A–D). Furthermore, integration with the TF-footprinting results suggests that differential expression of genes involved in these signaling pathways is likely driven by aberrant binding of transcription factors, including HNF1B, SOX10, and NFIC (Fig. 4A–D). A schematic example of the TGF-beta signaling pathway in INN-treated C15 cells on day 2 is shown in Supplementary Figure 5, which shows up- and down-regulated genes involved in the pathway (red and green labels, respectively). Overall, increases in motif-accessibility and up-regulation of specific TFs suggests that INN-mediated dysregulation of gene expression is primarily driven *via* aberrant TF-binding, ultimately leading to disruption of signaling pathways that are necessary for normal mesoderm formation.

## Discussion

In the present study, we demonstrate that INN exposure disrupts the mesoderm formation stage during cardiac differentiation of hESCs and hiPSCs. We found that a concentration of 25 nM INN is sufficient to disrupt mesoderm formation, and deleterious effects are most dramatic on mesoderm compared to endoderm and ectoderm lineages. It is worth noting that this concentration is significantly lower than levels observed in the blood of actual patients^19^. We further uncover potential molecular mechanisms that may underlie INN-induced disruption of differentiation, shedding light on factors that may cause developmental abnormalities that give rise to congenital heart defects, the leading form of birth defects observed in newborns each year.

Teratogenic potency and placental transfer efficiency of INN varies among different species; human was considered to be particularly sensitive to INN compared to mammals (e.g., monkey, rabbit, rat and mouse)^30^. It has been reported that INN exhibits an extended half-life (16 hours) in human body compared to all-*trans* RA (1 hour)^30,31^. Previous animal studies suggest that exogenous all-*trans* RAs and their metabolites (4-oxo-derivatives) showed higher teratogenicity than that of INN^32,33^. Moreover, INN exhibited lower activity in transactivation assays^34^, and its transcriptional regulation remains unclear due to low affinity for both RARs and RXRs^4^.

Conversely, based on genome-wide open-chromatin profiling of INN-treated cells during mesoderm differentiation, we discovered that INN-exposure induced expression and enhanced genomic DNA-binding of several TFs, including HNF1B, SOX10 and NFIC. We also identified genes likely regulated by these TFs are involved in important signaling pathways using computational biology methods. It is known that multiple signaling pathways are involved in the formation of the three germ layers (i.e., ectoderm, mesoderm, and endoderm), including Wnt/β-catenin, Notch, NODAL, BMP, Smad and FGF signaling^27,28^. It is also well-established that the mesodermal cells yield formation of the heart tube^35^. These mechanisms are also seen in mesoderm differentiation derived from human stem cells *in vitro*^23,26^.

Endogenous all-*trans* RA is known to regulate early mesodermal differentiation, neural development and patterning of the heart tube^3,36–41^. The enhanced protein expression of HNF1B, SOX10 and NFIC by INN exposure exhibited some similarity as seen in previous studies on RA-treatment, and these TFs play important roles in multiple differentiation process, including nephron, stomach development, beta cells and pancreatic precursor cells (HNFIB)^42–45^; osteoblast differentiation (NFIC)^46^; and neural crest differentiation (SOX10)^47,48^. The enriched GO terms of the INN-treated groups additionally reflect certain similarities with that of all-*trans* RA. For instance, up-regulated genes of the treatment groups are related to “neuron differentiation” (Supplementary Table 3), indicating that INN exerts effects similar to all-*trans* RAs so as to induce expression of genes involved in neural differentiation. However, no evidence of neuronal morphology was observed in INN-treated cells (Supplementary Figure 6). Thus, INN cannot be considered to be sufficient for neural development as endogenous RAs. In this study, we performed an *in-vitro* monolayer-differentiation method to evaluate impacts of INN on early mesodermal formation as well as ectoderm and endoderm. Embryonic development is more complex, thus validation for the interaction between INN, and these TFs and regulatory roles of these TFs, need to be addressed in future *in-vivo* studies.

Collectively, INN, as a 13-*cis*-RA, caused dysregulation of genes involved in signaling pathways underlying mesoderm differentiation *via* HNF1B, SOX10 and NFIC, leading to disruption in mesoderm formation. This study sheds new light on mechanistic studies in developmental toxicology by the use of human stem cells as an alternative system, and it also broadened our knowledge of mechanisms underlying congenital diseases of newborns due to maternal drug exposure during pregnancy at very low levels.

## Materials and Methods

### Chemicals

INN (Selleck Chemical LLC, TX) was prepared in dimethyl sulfoxide (DMSO), and then diluted to the final working concentration in medium for experiments.

### Cell culture and cell viability measurements

H1-hESC and C15-hiPSC lines were used in this study after obtaining them from the Stanford Cardiovascular Institute (SCVI) Biobank and the Stem Cell Core Facility of Genetics, Stanford University. The C15-hiPSC line was generated with lentivirus from skin fibroblasts from anonymous healthy persons. All pluripotent cell lines were grown in matrigel (Corning, CA)-coated 12-well plates in Essential 8™ Medium (Thermo Fisher Scientific, MA) at 37 °C in 5% CO_2_ in compressed air and high humidity. For cell viability tests, the H1 and C15 lines were placed in 24-well plates, and then exposed to graded concentrations (0–50 μM) of INN (in 0.1% DMSO as a vehicle control) from the second day for 48 h. The Celltiter-blue cell viability assay kit (Promega, WI) was used to measure cellular viability upon exposure to INN, and fluorescence intensity (excitation wavelength at 560 nm, emission at 590 nm) was recorded using a Tecan M1000 multimode plate reader (Tecan Systems, Inc., CA).

### Stem cell differentiation and developmental exposure

Differentiation of ectoderm and endoderm was performed in 24-well plates using a STEMdiff™ Trilineage Differentiation Kit (STEMCELL Technologies) according to the manufacturer’s instructions. Cardiomyocyte differentiation was performed in 12-well plates using a monolayer differentiation method with a PSC Cardiomyocyte Differentiation kit (Thermo Fisher Scientific) as described in our previous publication^18^. The cells were exposed to INN (in 0.1% DMSO) during the differentiation (Fig. 1A). We used 25 nM of INN as working concentration since we found it was a lowest-observed-adverse-effect level (LOAEL) of INN on cardiomyocyte differentiation (details see in Results). On days 0, 2, and 6 during cardiomyocyte differentiation period (before the medium was changed on that day), cells were collected using Accutase (Thermo Fisher Scientific). For each cell line and at each time-point, cells from two independent differentiation wells were used as two biologic replicates, and these collected cells were used for both RNA-seq and ATAC-seq experiments.

### RNA-seq and data analysis

We used two replicates from each cell line for the RNA-seq analysis. Total RNA was extracted using the miRNeasy Mini Kit (Qiagen, CA), and RNA was then subjected to DNase I digestion and purified using a RNeasy Mini spin column (Qiagen) according to the manufacturer’s instructions. RNA integrity was checked with a NanoDrop, and only samples with a 260/280 ratio between 2.0–2.1 were subsequently used for ribosom depletion. Purified RNA (2.5 μg) was used for ribosomal depletion using with the Ribo-Zero™ Gold Kit (Human/Mouse/Rat) (Epicentre Biotechnologies) according to the manufacturer’s instructions. The integrity of ribosome-depleted RNA was assessed with the Agilent RNA 6000 Pico Assay kit on the Agilent 2100 Bioanalyzer (Agilent Technologies). RNA-seq libraries were constructed using a ScriptSeq™ v2 RNA-Seq Library Preparation kit (Epicentre Biotechnologies, WI) according to the manufacturer’s instructions. The concentration of the library was measured with a Qubit Fluorometer (Thermo Fisher Scientific) and the size was determined using an Agilent High Sensitivity DNA kit on an Agilent 2100 Bioanalyzer. All RNA-seq libraries were sequenced using HiSeq. 4000 sequencers (Illumina) with 2 × 101 cycles. RNA-seq fastq data were aligned with Tophat^49^ (version 2.0.9) to the human hg19 reference genome. The human gene symbols and their raw counts were calculated using the HTSeq^50^. (version 0.6.1p1) package in Python with the hg19 *Homo sapiens* gtf file. Differential gene-expression analysis was performed using the edgeR package in R, and the normalization was performed using a trimmed mean of M-values (TMM) method across all samples^51^. In addition, Cufflinks (version 2.2.1) and GRCh37/hg19 Homo sapiens gtf file from UCSC Genome Browser were used to estimate transcripts abundance and generate their FPKM values. Hierarchical clustering analysis of transcriptome was performed using weighted gene co-expression network analysis (WGCNA)^20^. The Gene Ontology (GO) enrichment analysis was performed using DAVID on-line tool (version 6.8) (https://david.ncifcrf.gov/summary.jsp) and Gene Ontology Consortium (http://geneontology.org). The KEGG pathway^52–54^ analysis was performed using GAGE and Pathview^55,56^ packages in R.

### ATACseq and data analysis

We used two replicates from each cell line for the ATAC-seq analysis. The ATAC-seq protocol developed by Buenrostro *et al*.^57^ was used for profiling of chromatin landscape. 50,000 cells of each sample were collected and pelleted by centrifugation for 15 min at 500 g and 4 °C. Cell pellets were washed once with ice-cold 1x PBS and then pelleted again by centrifugation at the previous settings. Cell pellets were re-suspended in 25 μl of ice-cold lysis buffer (10 mM Tris-HCl, 10 mM NaCl, 3 mM MgCl_2_, and 0.1% Igepal CA-630, pH 7.4), and nuclei were pelleted by centrifugation for 30 min at 500 g and 4 °C. Supernatants were discarded and nuclei were re-suspended in 50 μl reaction buffer (2 μl of Tn5 transposase and 12.5 μl of TD buffer from a Nextera DNA Library Prep Kit from Illumina, and 22.5 μl nuclease-free H_2_O). The reaction was incubated at 37 °C for 30 min, and subsequently the reaction mixture was purified using MinElute PCR Purification Kit (Qiagen). The purified transposed DNA was amplified with NEBNext High-Fidelity 2 X PCR Master Mix (New England Biolabs) and custom-designed primers with barcodes^57^. Gel electrophoresis was used to remove primer dimers from the PCR products with 2% E-Gel EX Agarose Gels (Thermo Fisher Scientific), and then the PCR products were purified using QIAquick PCR Purification Kit (Qiagen). DNA concentration was measured with a Qubit Fluorometer (Thermo Fisher Scientific) and library sizes were determined using an Agilent High Sensitivity DNA kit on an Agilent 2100 Bioanalyzer. The ATAC-seq libraries were sequenced with a HiSeq. 2000 sequencer (Illumina) with 2 × 101 cycles, and the sequencing quality control was performed by the Stanford Center for Genomics and Personalized Medicine.

The raw data were then trimmed to remove the adapter sequences (CTGTCTCTTATACACATCT) with command-line tool cutadapt (1.8.1). The trimmed files were mapped to the human genome (hg19) using Bowtie2 (2.1.0) with default parameters. Read pairs, which aligned concordantly to the genome and had a mapping quality greater than 10, were kept for subsequent analysis. Mapping rate and mapped reads of the ATAC-seq data are shown in Supplementary Table 8. Read pairs mapped to mitochondrial DNA were discarded. Redundancy read pairs from PCR amplification were also removed afterward using Picard tools (version 1.79). Open accessible-regions for each library were defined by the peaks called by MACS2 (2.1.0) with the parameters “-g hs–nomodel–shift 0 –q 0.01”. Peaks located at blacklist genomics regions were removed using bedtools (2.25.0). These tracks show artifactual regions that tend to show artificially high signal and were identified by the ENCODE and modENCODE consortia^58^. The annotations of the peaks were achieved using ChIPpeakAnno, org.Hs.eg.db, and EnsDb.Hsapiens.v75 packages in R.

### Computational footprinting of TF-binding analysis

TF-footpinrting analysis was divided into two steps. The first step identifies sites throughout the genome where a motif is likely to be bound (*i*.*e*., footprint). The second step assigns a score to indicate differential binding or chromatin accessibility over footprints identified in the step 1 (differential footprint). We used merged bam files (mapped to gene coordinates) from each replicate to achieve enough sequencing depth for the footprinting analysis. Specifically, putative TF footprints were identified using the Protein Interaction Quantification (PIQ) footprinting algorithm^21^ against the JASPAR core vertebrate database (http://jaspar.genereg.net)^22^ of TF motifs as described in our previous publication^18^. Motifs matching the database of known TF target sequences were performed against the hg19 reference genome using the PIQ package pwmmatch.exact.r script. Afterwards, filtered ATAC-seq alignment reads were converted into binary RData files, and TF footprint scores were determined for each motif match using the PIQ package pertf.bg.r and common.r scripts with default settings. Putative TF footprints were filtered at a positive predictive value (PPV) cutoff of 0.7 (*i*.*e*., 70% of sites are bound after filtering) and, in each experiment the total number of binding events were then compared across conditions. In order to investigate relationships between TF-occupancy and gene regulation, the closest coding genes were assigned for each TF footprint/occupancy based on their transcription start sites (TSS) using *bedtools closest* command line of bedtools software (version 2.26.0). The differential footprint analysis was performed with Wellington-bootstrap algorithms^59^.

### Immunostaining

Immunostaining of three germ layers was performed using a Human Three Germ Layer 3-Color Immunocytochemistry Kit (R&D Systems). For immunostaining of cells during cardiomyocyte differentiation, the pluripotent cells were grown and differentiated in a glass-bottomed 12-well plate (MatTek, MA), and then cells were fixed and permeated in the plate using a Human Cardiomyocyte Immunocytochemistry Kit (Thermo Fisher Scientific). The primary antibodies included mouse anti-TRA-1-60 (ESI BIO, ST11016), rabbit anti-Nanog (Stemgent, 09-0020), goat anti-Brachyury (Fisher Sciences, AF2085), rabbit anti-Pax6 (BioLegend, 901301), rabbit anti-Nkx2-5 and mouse anti-Tnnt2 (Thermo Fisher Scientific, A25973). Secondary antibodies included goat anti-mouse IgG (Alexa Fluor 488 conjugated, Thermo Fisher Scientific, A10680), donkey anti-goat IgG (DyLight 488 conjugated, Thermo Fisher Scientific, SA5-10086), goat anti-rabbit IgG (H + L) Alexa Fluor 488 (Thermo Fisher Scientific, A-11034), and donkey anti-rabbit IgG (Alexa Fluor 594 conjugated, Thermo Fisher Scientific, A21207). The images were taken using Leica DMi8 Microsystems and Zeiss LSM710 inverted confocal microscope, and then the images were processed using the Fiji software.

### Flow cytometry and analysis

Differentiating cells (50,000 cells) on day 2 were collected for flow cytometry. The cells were washed with DPBS buffer (Thermo Fisher Scientific), and then were fixed and permeabilized using Cytofix/Cytoperm (BD Biosciences). Afterwards, the cells were labeled with mouse anti-Brachyury (Abcam, ab140661) and goat anti-mouse IgG (Abcam, Alexa Fluor 488 conjugated, ab150117). In addition, mouse Otx2 Alexa Fluor® 488-conjugated antibody (R&D Systems, IC1979G-025) and mouse SOX17 PE-conjugated antibody (R&D Systems, IC19241P) were used for and ectoderm and endoderm, respectively. Ice-cold DPBS (with 1% FBS) was used as the flow cytometry buffer for re-suspending cells. Flow cytometry was performed using a FACSAria II cytometer (BD Biosciences). The data were analyzed using FlowJo software (version 10.1).

### Real-time QPCR

cDNA was synthesized from 1 μg of total RNA using an SuperScript VILO cDNA Synthesis Kit (Thermo Fisher Scientific) following the manufacturer’s protocol. 18S rRNA was used as a normalizing gene. PCR amplification was performed with a QuantStudio™ 6 Flex Real-Time PCR System (Thermo Fisher Scientific) in 20-μl reactions using 1 μl of cDNA (10 ng of total input RNA), 200 nM of each forward and reverse primer and 1X PowerUp SYBR™ Green Master Mix (Applied Biosystems). The real-time PCR program consisted of 1 cycle of 95 °C for 5 min; and 40 cycles of 95 °C for 15 s, 58 °C 30 s and 72 °C for 30 s. Relative gene expression data were calculated using the ΔΔCt method^60^. The primer sequences for *NFIC* are^61^: forward, 5′-GGACAGGGATGGGCTCTG-3′; reverse, 5′-CGTTCTTCTGAGGCCAGTGC-3′. The primer sequences for *SOX10* are^62^: forward, 5′-GACCAGTACCCGCACCTG-3′; reverse, 5′-CGCTTGTCACTTTCGTTCAG-3′. 18S rRNA was used as a normalizing gene, and the primer seuqneces are^63^: forward, 5′-GCAATTATTCCCATGAACG-3′; reverse, 5′-GGGACTTAATCAACGCAAGC-3′.

### Western blot

The cells were harvested in RIPA lysis buffer (EMD Millipore, CA) contain one tablet of Pierce™ protease and phosphatase inhibitor (Thermo Fisher Scientific), and the proteins were purified using a Branson Digital Sonifier homogenizer (Branson Ultrasonics). 15 μg of protein from each sample was separated on NuPAGE 4–12% Bis-Tris protein gels (Thermo Fisher Scientific) and transferred to nitrocellulose membranes (Thermo Fisher Scientific). The protein-bound membranes were blocked with 5% of blotting-grade blocker (Bio-Rad) in PBST for one hour at room temperature and incubated with a primary antibody in 5% of blotting-grade blocker in PBST overnight at 4 °C. After washing with PBST buffer, the membranes were incubated with horseradish peroxidase (HRP)-conjugated-secondary antibody for 1 h at room temperature. The membranes were developed with SuperSignal West Femto Maximum Sensitivity Substrate (Thermo Fisher Scientific) and exposed on a ChemiDoc Touch imaging system (Bio-Rad) for imaging. The primary antibody used in this study included rabbit anti-HNF1B (Abcam, ab128912), rabbit anti-SOX10 (Bethyl Laboratories, A305-521A-T), rabbit anti-NFIC (Bethyl Laboratories, A303-123A-T). The secondary antibody was HRP-conjugated-goat anti-rabbit IgG (GE Healthcare, RPN4301). Mouse anti-beta actin (horseradish Peroxidase [HRP] conjugated) (Invitrogen, MA515739HRP) was used as the reference.

## Electronic supplementary material

Supplementary video 1. Cardiomyocytes derived from C15-hiPSC

Supplementary video 2. Cardiomyocytes derived from H1-hESC

Supplementary video 3. Cardiomyocytes derived from C15-hiPSC which was pre-treated with 25nM of INN

Supplementary video 4. Cardiomyocytes derived from H1-hESC which was pre-treated with 25nM of INN

Supplementary information

Supplementary Table 1

Supplementary Table 2

Supplementary Table 3

Supplementary Table 4

Supplementary Table 5

Supplementary Table 6

Supplementary Table 7

Supplementary Table 8

## Data Availability

Both RNA-seq and ATAC-seq data generated for this work have been deposited in NCBI’s Gene Expression Omnibus (GEO), and they are accessible through GEO SuperSeries accession number GSE85881: GSE85880 for the RNA-seq, and GSE85879 for the ATAC-seq.
