# Supplementary material for: Disruption of mesoderm formation during cardiac differentiation due to developmental exposure to 13-cis-retinoic acid
Source: Sci Rep. 2018 Aug 28;8:12960. doi: 10.1038/s41598-018-31192-0 (PMC6113333; doi:10.1038/s41598-018-31192-0)

SUPPLEMENTARY INFORMATION

Disruption of mesoderm formation during cardiac differentiation due to developmental exposure to 13-*cis*-retinoic acid

Qing Liu[1], Kevin Van Bortle[1], Yue Zhang[1,2], Ming-Tao Zhao[3], Joe Z Zhang[3], Benjamin S Geller[1], Joshua J Gruber[1,4], Chao Jiang[1], Joseph C Wu[3], Michael P Snyder[1]

[1]Department of Genetics, Stanford University School of Medicine, Stanford, California 94305, USA.

[2] Genetics Bioinformatics Service Center, Stanford University School of Medicine, Stanford, California 94304, USA

[3]Stanford Cardiovascular Institute, Stanford University School of Medicine, Stanford, California 94305, USA.

[4]Department of Medicine, Oncology Division, Stanford University School of Medicine, Stanford, California 94305, USA.

Corresponding author: Michael P Snyder, mpsnyder@stanford.edu
Department of Genetics, 300 Pasteur Drive, M-344A,
Stanford University School of Medicine,
Stanford, California 94305, USA

SUPPLEMENTARY INFORMATION

Legends

Supplementary video 1. Cardiomyocytes derived from C15-hiPSC

Supplementary video 2. Cardiomyocytes derived from H1-hESC

Supplementary video 3. Cardiomyocytes derived from C15-hiPSC which was pre-treated with 25nM of INN

Supplementary video 4. Cardiomyocytes derived from H1-hESC which was pre-treated with 25nM of INN

Supplementary Table 1. Differentially expressed genes with their fold changes and FDR at each condition.

Supplementary Table 2. Top 10 enriched GO terms (BP) of dysregulated genes in control cells derived from H1 and C15 on day 2 and day 6.

Supplementary Table 3. Top 10 enriched GO terms (BP) of dysregulated genes in INN-treated cells derived from H1 and C15 on day 2 and day 6.

Supplementary Table 4. Significantly enriched GO terms (FDR<0.05) of overlapping up- and down-regulated genes between H1 and C15.

Supplementary Table 5. Differential footprint analysis results. Putative TF footprints were identified using the Protein Interaction Quantification (PIQ) footprinting algorithm against the JASPAR core vertebrate database of TF motifs. And the differential footprint analysis was performed with Wellington-bootstrap algorithms. The values in the table represent the log2-transferred ration of binding events between INN and control groups on day 2.

Supplementary Table 6. List of genes that were predicted to be regulated by HNF1B, SOX10 and NFIC. Genes were selected by positive predicting value larger than 0.7 (PPV>0.7) from footprinting analysis of ATAC-seq data and their FDR values less than 0.5 (FDR<0.05) from gene expression analysis of RNA-seq data.

Supplementary Table 7. Statistically enriched KEGG pathways in cells derived from H1 and C15 on day 2.

Supplementary Table 8. Mapping rate and mapped reads of ATAC-seq data used for the analysis.

SUPPLEMENTARY INFORMATION

Supplementary Figure 1. Immunostaining of cardiomyocyte progenitors with TNNT (green) and NKX2-5 (red) on day 6. No cardiomyocyte progenitors were found in INN-treated cells. The nuclei were stained with DAPI (blue).

Supplementary Figure 2. Immunostaining of ectoderm and endoderm derived from H1-ESCs upon exposure to INN. Ectodermal and endodermal cells were detected with Otx-2 and SOX17 antibodies, respectively. 25 nM of INN caused minor decreases in Otx-2$^+$-cells in ectoderm and moderate decrease in SOX-17$^+$-cells in endoderm cells, respectively.

Supplementary Figure 3. Flow cytometry analyses of ectoderm and endoderm derived from H1-ESCs upon exposure to INN. Ectodermal and endodermal cells were detected with 488-conjugated Otx-2 and PE-conjugated SOX17 antibodies, respectively. 25 nM of INN caused decrease in SOX-17$^+$-endoderm cells, but no significant decreases in Otx-2$^+$- ectoderm cells was observed.

Supplementary Figure 4. INN exposure induced gene expression of NFIC and SOX10. Barchart represents relative expression levels of NFIC and SOX10 in both C15-hiPSC and H1-ESC upon exposure to INN at day2. Relative expression levels were compared to that in control after normalization against 18S rRNA. T-test were perform for statistical analysis, * $p<0.05$.

Supplementary Figure 5. KEGG view of TGF-beta signaling pathway based on the gene expression data of INN-treated cells of C15 on day2. The figure was created using the gage package in R with KEGG pathway[1-3] map04350 (https://www.genome.jp/dbget-bin/www_bget?map04350), and red and green represent the up- / down-regulation of genes upon INN exposure, respectively.

Supplementary Figure 6. Immunostaining of INN-treated cells on day 6 with neuronal maker Pax6. No Pax6$^+$-cells were detected in day6 cells differentiated from H1 upon exposure to INN.

Supplementary Figure 7. Full-length blots used for the cropped version of Figure 3F are shown. A. Staining of HNF1B in INN-treated and control cells at day2 and day6. HNF1B was not detected in control cells. Data of day6 were not used in Figure 3F. **B.** Staining of SOX10 in INN-treated and control cells of H1 and C15, two blots were performed for each cell line. **C.** Staining of NFIC in INN-treated and control cells of H1 and C15. **C.** Staining of NFIC in INN-treated and control cells of H1 and C15.

Reference

Kanehisa, M., Furumichi, M., Tanabe, M., Sato, Y. & Morishima, K. KEGG: new perspectives on genomes, pathways, diseases and drugs. Nucleic Acids Res. 45, D353-D361 (2017).

SUPPLEMENTARY INFORMATION

Kanehisa, M., Sato, Y., Kawashima, M., Furumichi, M. & Tanabe, M. KEGG as a reference resource for gene and protein annotation. Nucleic Acids Res. 44, D457-D462 (2016).

Kanehisa, M. & Goto, S. KEGG: Kyoto Encyclopedia of Genes and Genomes. Nucleic Acids Res. 28, 27-30 (2000).

Supplementary Figure 1

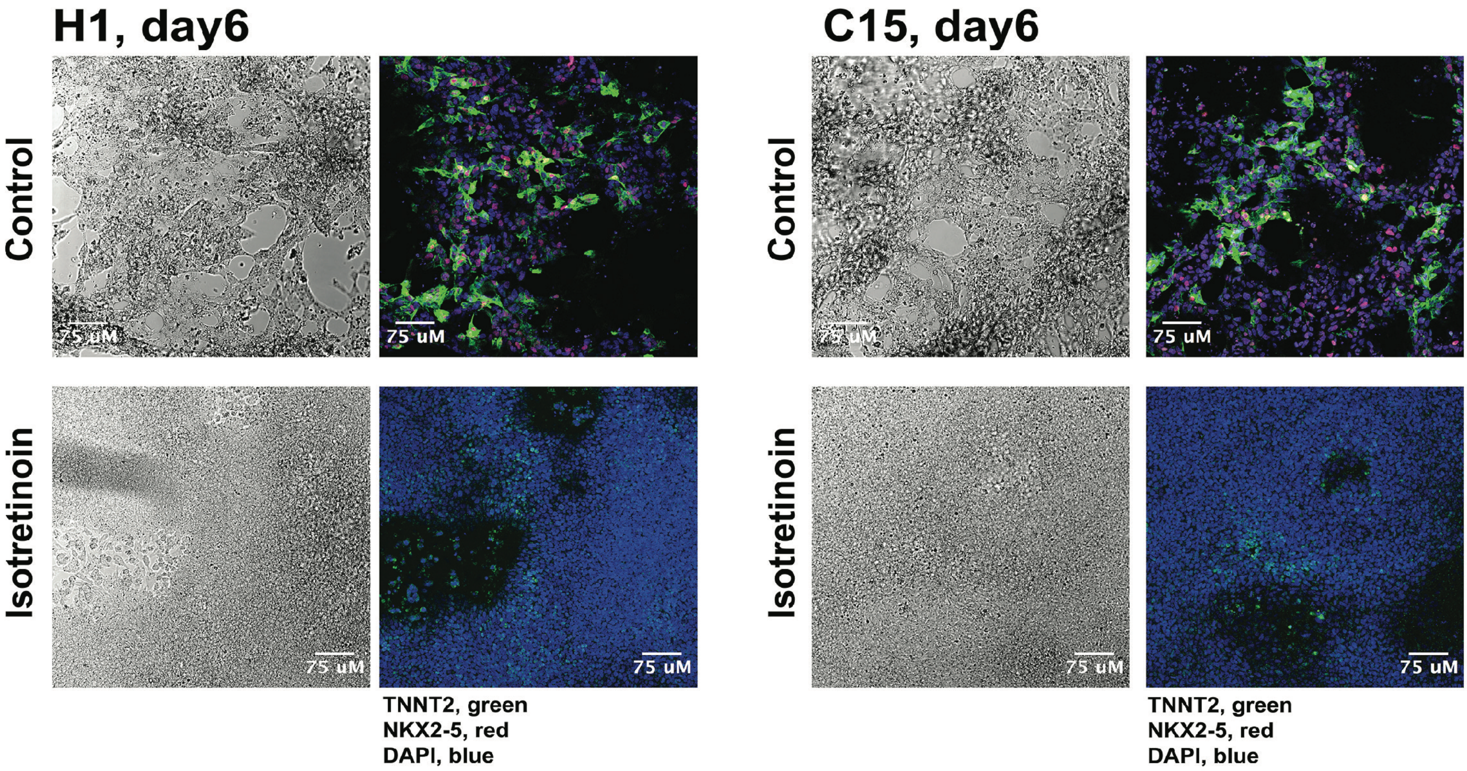

Supplementary Figure 2

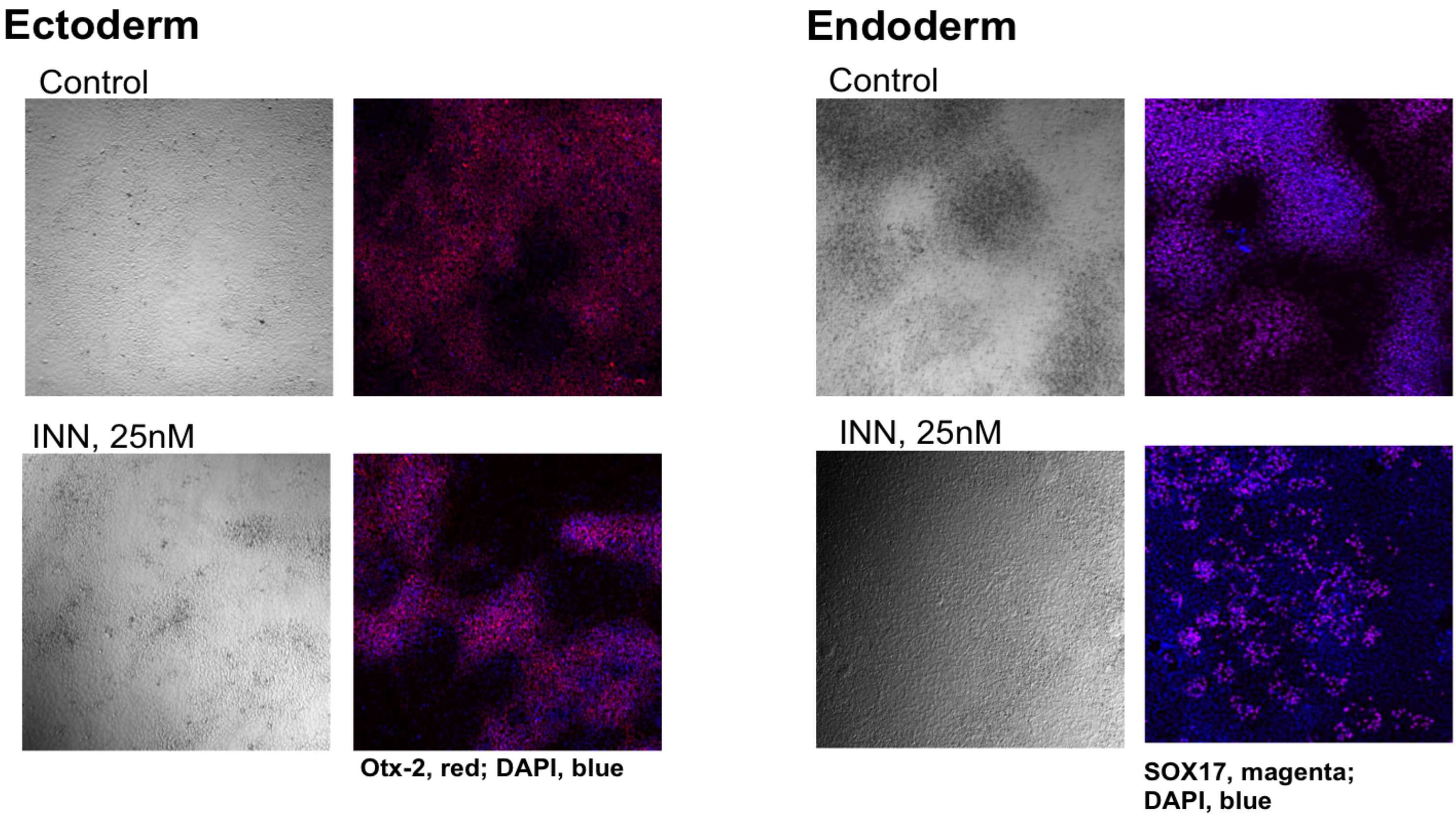

Supplementary Figure 3

Ectoderm

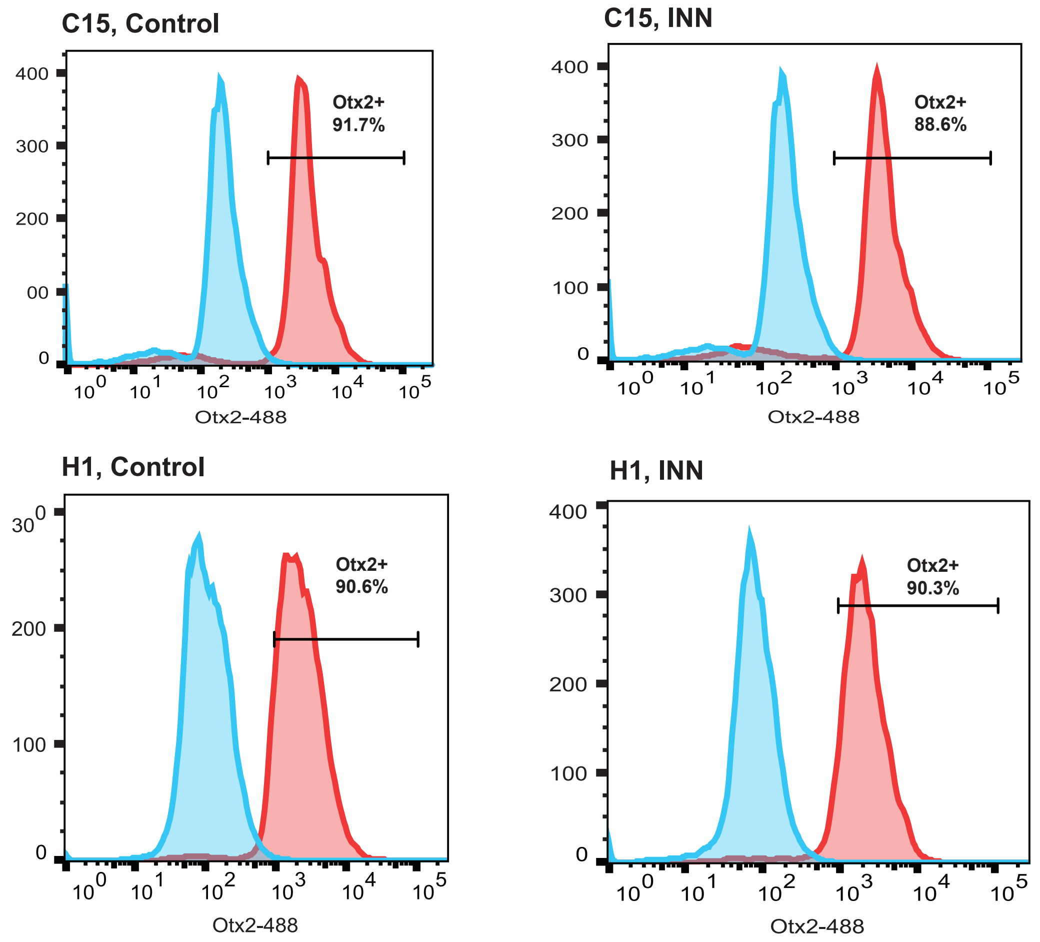

Endoderm

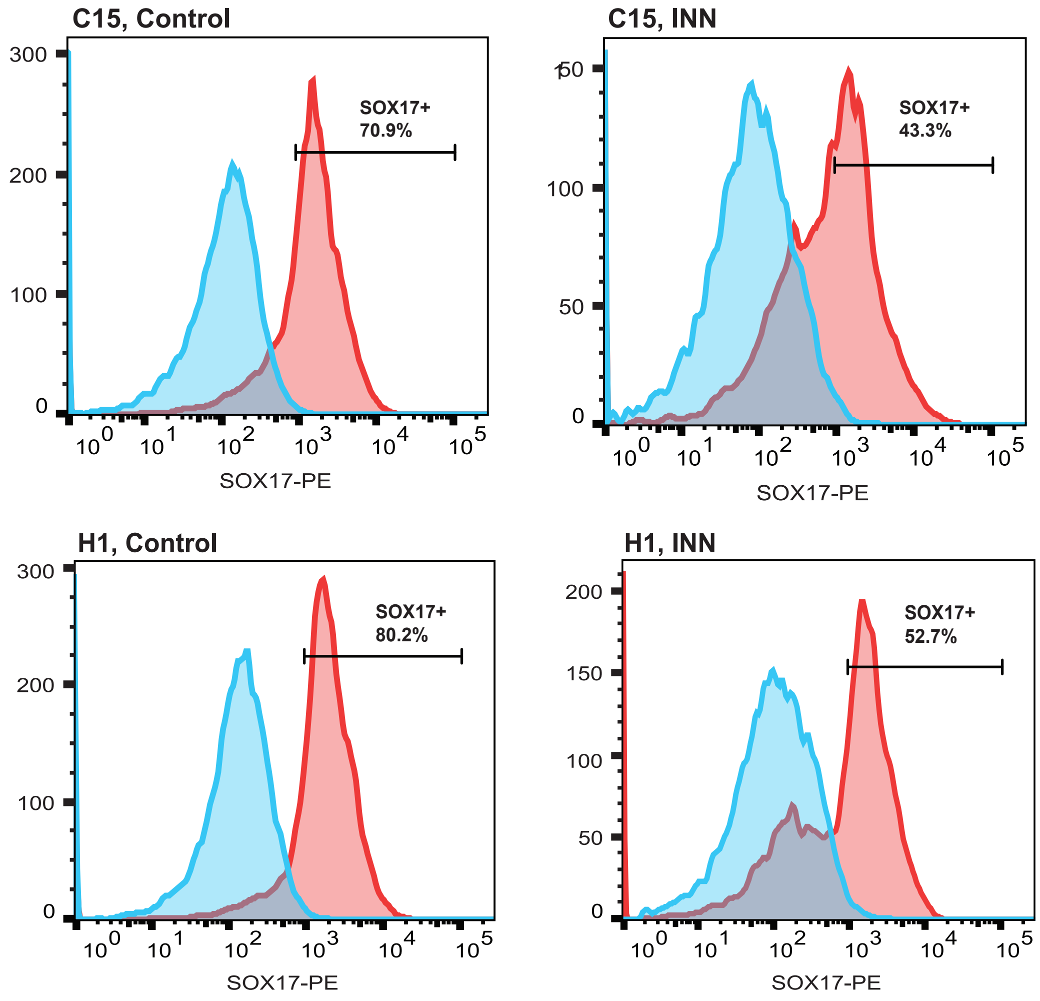

Supplementary Figure 4

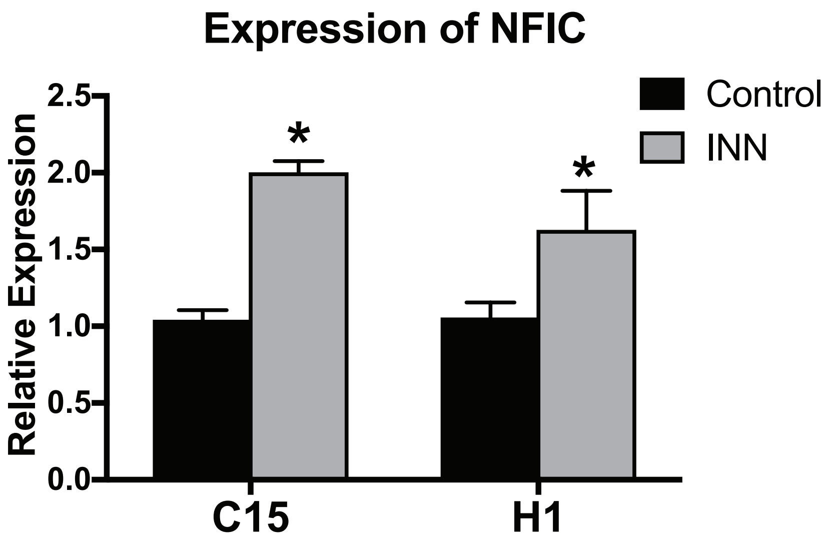

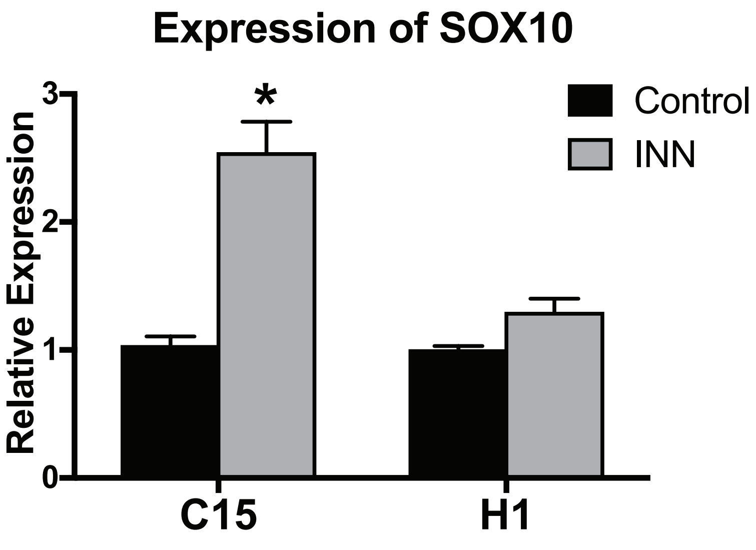

Supplementary Figure 5

C15, INN-treat vs Control, day2

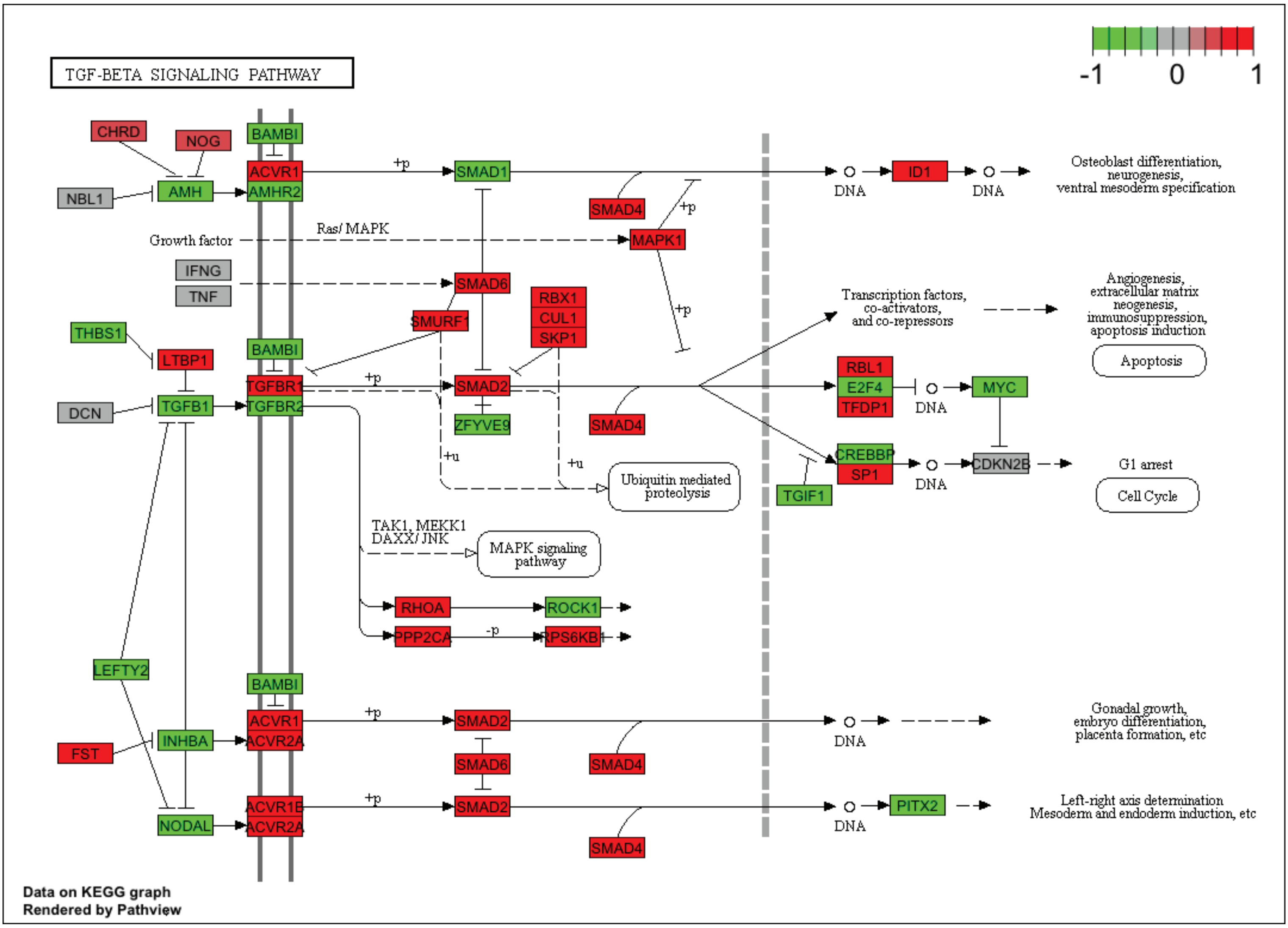

Supplementary Figure 6

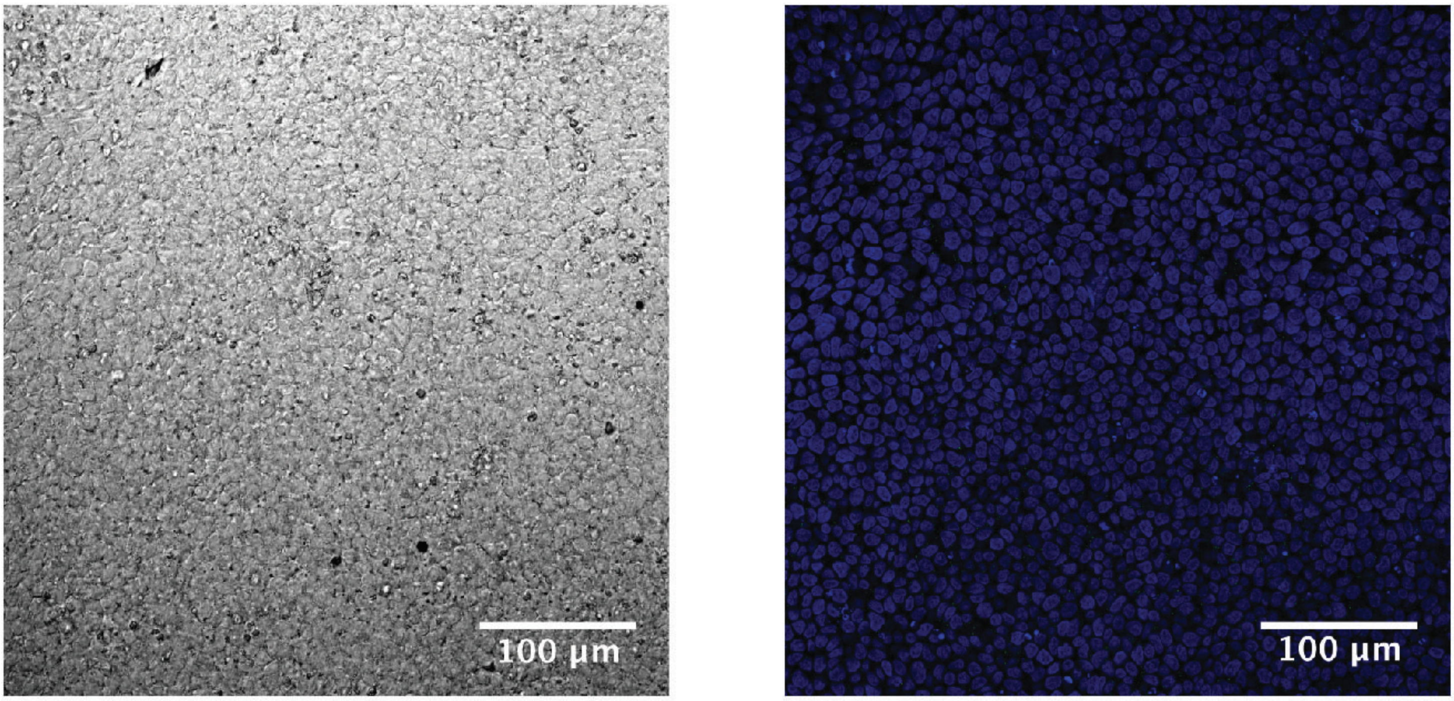

Pax6, green; DAPI, blue

Supplementary Figure 7

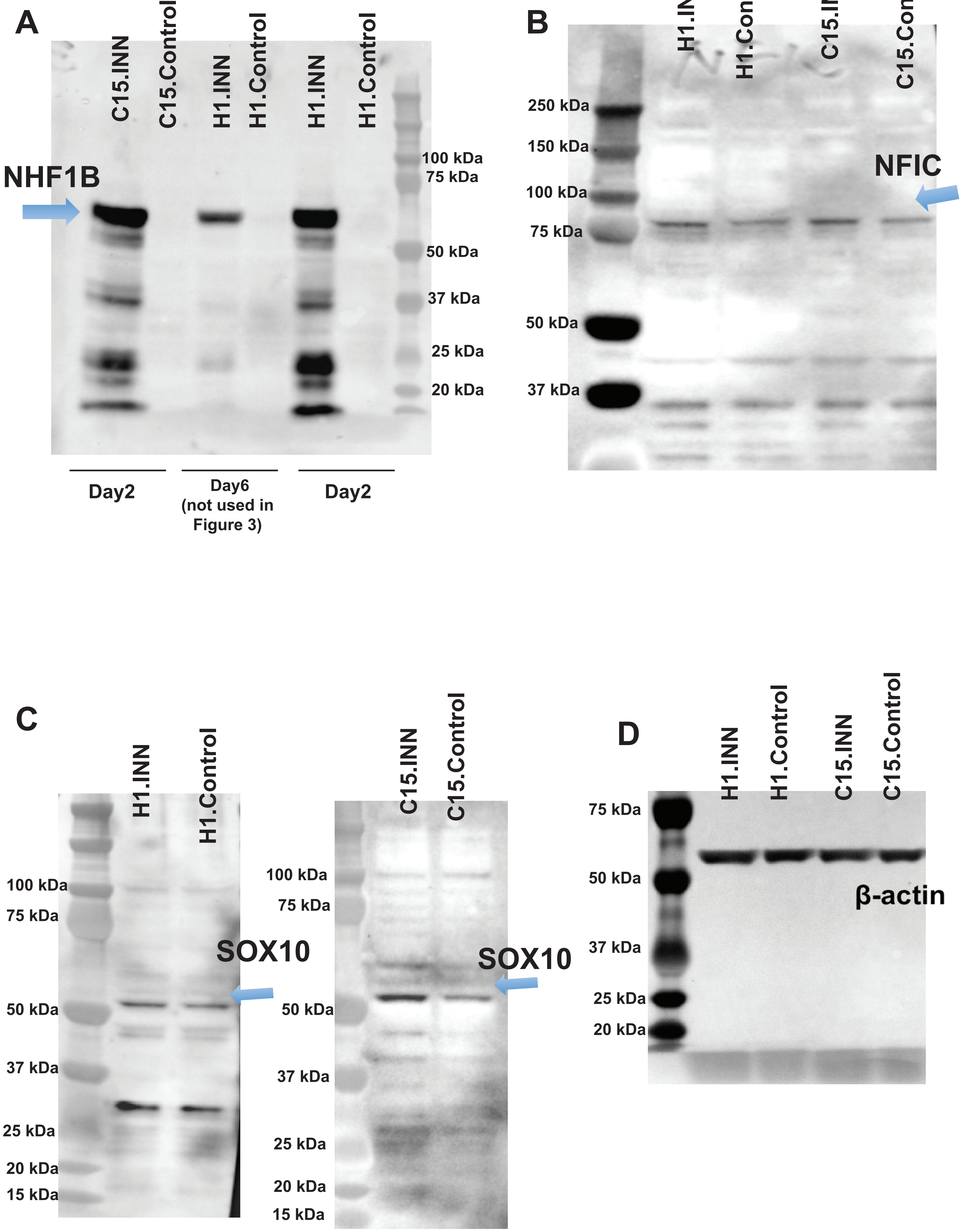

Supplement: Supplementary file 5 — Supplementary information [file 41598_2018_31192_MOESM5_ESM.pdf]
